# New Multiscale Characterization Methodology for Effective Determination of Isolation–Structure–Function Relationship of Extracellular Vesicles

**DOI:** 10.3389/fbioe.2021.669537

**Published:** 2021-06-07

**Authors:** Thanh Huyen Phan, Shiva Kamini Divakarla, Jia Hao Yeo, Qingyu Lei, Priyanka Tharkar, Taisa Nogueira Pansani, Kathryn G. Leslie, Maggie Tong, Victoria A. Coleman, Åsa Jämting, Mar-Dean Du Plessis, Elizabeth J. New, Bill Kalionis, Philip Demokritou, Hyun-Kyung Woo, Yoon-Kyoung Cho, Wojciech Chrzanowski

**Affiliations:** ^1^Sydney School of Pharmacy, Faculty of Medicine and Health, Sydney Nano Institute, The University of Sydney, Camperdown, NSW, Australia; ^2^School of Chemistry, The University of Sydney, Camperdown, NSW, Australia; ^3^Department of Dental Materials and Prosthodontics, Araraquara School of Dentistry, UNESP-Universidade Estadual Paulista, Araraquara, Brazil; ^4^Nanometrology Section, National Measurement Institute Australia, Lindfield, NSW, Australia; ^5^School of Chemistry, Faculty of Science, Sydney Nano Institute, The University of Sydney, Camperdown, NSW, Australia; ^6^Maternal-Fetal Medicine Pregnancy Research Centre, The Royal Women’s Hospital, and Department of Obstetrics and Gynaecology, The University of Melbourne, Parkville, VIC, Australia; ^7^Department of Environmental Health, Center for Nanotechnology and Nanotoxicology, Harvard T.H. Chan School of Public Health, Boston, MA, United States; ^8^Center for Soft and Living Matter, Institute for Basic Science (IBS), Ulsan, South Korea; ^9^Department of Biomedical Engineering, Ulsan National Institute of Science and Technology (UNIST), Ulsan, South Korea

**Keywords:** extracellular vesicles, mesenchymal stromal/stem cell, isolation methods, ultracentrifugation, tangential flow filtration, nanodosimetry, single vesicle analysis, exosomes

## Abstract

Extracellular vesicles (EVs) have been lauded as next-generation medicines, but very few EV-based therapeutics have progressed to clinical use. Limited clinical translation is largely due to technical barriers that hamper our ability to mass produce EVs, i.e., to isolate, purify, and characterize them effectively. Technical limitations in comprehensive characterization of EVs lead to unpredicted biological effects of EVs. Here, using a range of optical and non-optical techniques, we showed that the differences in molecular composition of EVs isolated using two isolation methods correlated with the differences in their biological function. Our results demonstrated that the isolation method determines the composition of isolated EVs at single and sub-population levels. Besides the composition, we measured for the first time the dry mass and predicted sedimentation of EVs. These parameters were likely to contribute to the biological and functional effects of EVs on single cell and cell cultures. We anticipate that our new multiscale characterization approach, which goes beyond traditional experimental methodology, will support fundamental understanding of EVs as well as elucidate the functional effects of EVs in *in vitro* and *in vivo* studies. Our findings and methodology will be pivotal for developing optimal isolation methods and establishing EVs as mainstream therapeutics and diagnostics. This innovative approach is applicable to a wide range of sectors including biopharma and biotechnology as well as to regulatory agencies.

## Introduction

Current medicine has only taken us so far in reducing disease and the tissue damage that it causes. Extracellular vesicles (EVs) have been hailed as the next generation of medicines. EVs are membrane-surrounded nanoscale structures secreted ubiquitously by cells. They contain multiple substances that influence the function of surrounding cells (Lötvall et al., 2014; Iraci et al., 2016). Since EV composition reflects the composition of the parent cell, EVs are ideal candidates for use in disease diagnosis (Candelario and Steindler, 2014). EVs are already considered as diagnostic biomarkers for cancer, cardiovascular, neurodegeneration, and kidney diseases (Taylor and Gercel-Taylor, 2008; Candelario and Steindler, 2014; Danielson and Das, 2014). It is also well established that EVs transfer numerous molecules including proteins, lipids, and nucleic acids between cells, and these molecules can act synergistically and influence the behavior of surrounding cells (Yanez-Mo et al., 2015). Since EVs can deliver multiple molecules to reprogram the injured cells and mediate the de-differentiation of cells, EVs can be used for tissue repair/regeneration (Guo et al., 2011). EVs derived from stem cells are recognized as “second generation” stem cell therapies (Candelario and Steindler, 2014; György et al., 2015)—made by cells for cells. Compared with stem cells, EVs have key advantages including low immunogenicity, no ability to self-replicate (no risk of cancer), high resistance to hostile environments, and improved bioactivity and stability upon storage (Piffoux et al., 2017). However, despite all these potential advantages, very few EV applications have progressed to clinical use (Yekula et al., 2020).

The limited clinical translation of EVs is largely due to technical barriers that hamper the mass production of EVs, i.e., the ability to isolate, purify, and characterize them effectively at single vesicle (nano), sub-population, and population levels (Ramirez et al., 2018). Since EV sizes range from 50 to 150 nm and they are secreted into rich multicomponent media or body fluids, isolation and characterization are not trivial and remain as key challenges in the field (Ramirez et al., 2018). The molecular corona, which cloaks EVs and is likely to cover some of the surface markers, adds to the complexity of these challenges (Simonsen and Munter, 2020). Since the corona changes the physicochemical characteristics of EVs and their affinity to the substrates used in some isolation methods, it is difficult to isolate and characterize EVs effectively. Moreover, EV isolates often contain lipoproteins, protein aggregates, and non-vesicle macromolecules (Sunkara et al., 2016). We also know that cell-free DNA can adsorb to lipid nanoparticles (Gardner et al., 2020), which is likely to occur for circulating EVs too, but surprisingly, this phenomenon is largely overlooked in the field. These “contaminants” influence the biological function of EVs and are not trivial to detect due to the sensitivity of experimental methods. This also means that it is difficult to decouple them from the isolated EV populations (Mateescu et al., 2017). However, these contaminants could potentially work synergistically with EVs to achieve specific therapeutic function in the body (Thery et al., 2018). Therefore, for practical utilization of isolation protocols, it is necessary first to perform comprehensive physicochemical and molecular characterization of EV isolates at sub/population and single vesicle levels and to measure functional responses to EVs in adequate cell/animal-based models.

The most commonly used approach to isolate EVs is ultracentrifugation, which involves multistep differential centrifugations to pellet vesicles (Ismail et al., 2013; Gardiner et al., 2016). However, ultracentrifugation is labor intensive, requires large sample volumes, and produces a relatively low yield of enriched EVs (Kang et al., 2017). Numerous alternative isolation methods have been developed including density gradients (DG) and size exclusion chromatography (SEC). Although DG and SEC usually result in high-purity EV, these protocols are time consuming, characterized by poor yields and suitable for small input volumes only (less than 5 ml) (Witwer et al., 2013; Coumans et al., 2017). Immunoaffinity-based approaches can also be used for EV isolation (Li et al., 2019). In these approaches, EVs are “collected” by beads functionalized with EV-specific antibodies; thus, the isolation process is solely related to affinity of EVs to selected antibodies. The collection of EVs is based on the assumption that specific markers are present on the surface of EVs. This means that these approaches are highly selective and likely to isolate only some fractions of EV populations. More importantly, these methods fail to account for the aforementioned corona making these approaches even more selective (Simonsen and Munter, 2020). Furthermore, at this stage, only small quantities of biological samples can be processed in immunoaffinity-based isolation (Momen-Heravi et al., 2013). In contrast, tangential flow filtration (TFF) is capable of processing scalable volumes of biological fluids and producing high EV yield (Busatto et al., 2018). TFF is technically simple to operate and requires low-cost instrumentation. These features make TFF well suited to isolate EVs at large scale. However, to enable broader applications of TFF, its advantages in comparison with ultracentrifugation (and other methods) must be determined.

Previous studies investigated the differences in physical properties between EVs isolated using TFF and ultracentrifugation (Busatto et al., 2018; Heath et al., 2018). However, the results are limited to selected physical characteristics of EVs (e.g., yield, size distribution, morphology, and surface markers) and do not show their correlation with biological effects. To the best of our knowledge, there is no available studies investigating how the physicochemical properties of EVs contribute to the EV functionality. Here, using a combination of high-resolution optical and non-optical techniques, as well as functional assays, we interrogated the differences between EVs isolated using ultracentrifugation and TFF at single vesicle, sub-population, and population levels. The significance of this work is in a new methodology, which enables characterizing EVs and demonstrating how the isolation methods influence the physicochemical/molecular composition of EVs and functional cell responses to EVs. For the first time, we measured the dry mass of large EVs or EV agglomerates (>100 nm), predicted the sedimentation of EVs, and developed a new fluorescent probe to assess the functionality of EVs. The key strength of this study lies in the comprehensive characterization of EVs at single vesicle, EV sub-population, and EV population levels, as well as in the analysis of cellular responses (i.e., EV uptake) at single-cell level. To achieve desired statistical and scientific validation of our approach, we used two cell types, which produce different quantities of EVs and that these EVs characterize with different molecular cargo; specifically, we used chorionic and decidual mesenchymal stem cells (CMSC29 and DMSC23, respectively) (Qin et al., 2016; Kim et al., 2019a).

Our study provides evidence that EVs’ physicochemical characteristics (at single vesicle, subpopulation, and population level) as well as their biological function, depends on the isolation method. The differences in physicochemical properties of EVs were shown to correlate well with the functional effects of EVs. We concluded that isolation method determines the composition of EV isolates. These findings are of critical significance in the field because they suggest that isolation method is pivotal in establishing downstream applications of EV as diagnostic biomarkers, therapeutics, and in fundamental biology. Notably, this work highlights the importance of nanoscale and single-particle characterization methods in EV research and the need for the integrated use of physicochemical and functional assays.

## Materials and Methods

### Cell Culture and Maintenance

Both chorionic and decidual MSCs cell lines (CMSC29 and DMSC23) were obtained from the Royal Women’s Hospital in Melbourne, Australia. Telomerase reverse transcriptase (hTERT) was transduced into primary MSCs from the fetal chorion and maternal decidual components of human placenta to create the CMSC29 and DMSC23 cell lines, respectively (Qin et al., 2016). Since CMSC29 and DMSC23 were derived from different parts of the placenta, they are exposed to different levels of oxidative stress (Kusuma et al., 2016); thus, they required different types of medium. CMSC29 cells were cultured in 85% AmnioMAX^TM^ C-100 basal medium and 15% AminoMAX^TM^ C-100 supplement (Invitrogen^TM^, ThermoFisher Scientific). DMSC23 cells were cultured in MesenCult^TM^ MSC basal medium (Human), 10% Mesenchymal stem cell stimulatory supplement (STEMCELL Technologies, Canada), GlutaMAX^TM^ (Life Technologies, Australia), and antibiotics (Pen/Strep) (100 U of penicillin and 0.1 mg/ml of streptomycin, Sigma-Aldrich, Australia). BEAS-2B cells were cultured in medium containing Dulbecco’s modified Eagle’s medium (DMEM medium-high glucose, Sigma-Aldrich, Australia) supplemented with 10% fetal bovine serum (FBS, Bovogen, Australia), and antibiotics (Pen/Strep). Cells were sub-cultured every 2–3 days and maintained in the incubator at 37°C supplemented with 5% CO_2_. Hanks’ balanced salt solution (HBSS, Sigma-Aldrich, Australia) was used for washing CMSC29 and DMSC23 cells. TrypLE^TM^ Express (Gibco, Denmark) was used to dissociate the adherent cells. EV isolation and collection were from CMSC29 and DMSC23 cells at passages P23–28.

### Isolation of Extracellular Vesicles by Ultracentrifugation

CMSC29 and DMSC23 cells were cultured to 80% confluency. Cells were washed twice with HBSS before incubating cells with EV isolation media (MesenCult^TM^ MSC basal medium) containing 0.5% (w/v) bovine serum albumin (BSA, Sigma-Aldrich, Australia) for 48 h. After 48 h, EV-containing media was collected and centrifuged at 500 × *g* for 5 min and 2,000 × *g* for 10 min to remove cells and debris. The supernatant was then transferred to thick-wall polycarbonate ultracentrifuge tubes (Seton Scientific Inc, United States) and centrifuged at 100,000 × *g* for 60 min at 4°C using rotor Ti-70 in an Optima LE-80K Ultra Centrifuge (Beckman Coulter, Australia). The harvested EV pellet was resuspended in 1 ml of RNase-free phosphate-buffered saline (RNase-free PBS, Lonza, Australia) and washed using ultracentrifugation at 100,000 × *g* for 60 min at 4°C. The pellet was resuspended in 1 ml of RNase-free PBS and transferred to RNase-free microcentrifuge tubes. The EV pellets were stored at 4°C to avoid losing biological function during the freezing process.

### Isolation of Extracellular Vesicles by Tangential Flow Filtration

After removing cells and debris by centrifugation at 500 × *g* (5 min) and 2,000 × *g* (10 min) as described above, the EV containing supernatant was filtered (0.45 μm) and transferred to TFF-Easy 20-nm pores (HansaBioMed/Lonza, Tallinn, Estonia) for EV concentration. The EV concentration process was described in the manufacturer’s protocol (HansaBioMed/Lonza), and EVs were finally diafiltrated in RNase-free PBS.

### Size and Concentration Measurement Using Nano-Flow Cytometry

The size and concentration of EVs were measured using NanoFCM (Xiamen Fuliu Biological Technology Co., Ltd, Xiamen, China). A mixture of silica nanospheres (68, 91, 113, and 155 nm) was used as the size standard for the construction of a calibration curve and standard 200-nm polystyrene spheres were used for laser alignment. All events were collected for 120 s, and size (SSC) triggering was used to detect EVs. The total events collected ranged from 3,000 to 6,000 events. The representative histogram was conducted from triplicate measurements of each EV sample.

### Size and Concentration Measurement Using Particle Tracking Analysis

EV samples were diluted with RNase-free water to achieve a concentration between 1 × 10^8^ and 1 × 10^9^ EVs/ml and measured using a NanoSight NS300 (Malvern Panalytical Ltd, Malvern, United Kingdom). A syringe pump with a speed of 40 μl/min was used, and cell temperature was set at 25°C. Embedded laser wavelength was 488 nm, and the particles were imaged with an auto-focus camera for 60 s. Data were obtained at camera-level 11. The analysis settings were set to “auto,” and the detection threshold was set to 5 in the NanoSight Software NTA (version 3.2) to assess mean and modal particle diameters, D50 values (which represents the 50th percentile of the averaged cumulative number-weighted particle size distribution) and particle number concentration. For each EV sample, three repeat measurements were conducted.

### Size Measurement Using Dynamic Light Scattering

EV samples were diluted in RNase-free water to achieve a particle concentration ranging from 1 × 10^9^ to 1 × 10^10^ EVs/ml and were measured in a Zetasizer Ultra (Malvern Panalytical Ltd, Malvern, United Kingdom). The manufacturer’s default software setting for EVs (liposomes) was selected, and three cycles were performed for each measurement at 4°C. Data were analyzed using general purpose mode in ZS XPLORER software (version 1.2.0.91), and the size distribution of EV populations was presented as a percentage of intensity. Three consecutive runs were performed for each sample.

### Size and Concentration Measurement Using Tunable Resistive Pulse Sensing

TRPS was performed using a qNano (IZON, New Zealand) to measure the particle size and the concentration of EVs. EVs were suspended in electrolytes and passed through an engineered pore (NP100), which provided direct measurement of size and concentration. Buffers and reagents were freshly prepared and filtered (0.22 μm) before the measurement. The detailed protocol for qNano measurements was described in the study by Vogel et al. (2011). Three independent measurements were done for each individual EV sample.

### Morphology Analysis of Extracellular Vesicles Using Atomic Force Microscopy

EV samples were placed onto zinc selenide prism, dried overnight, and subsequently imaged using atomic force microscopy (AnasysInstruments, United States). Images were obtained in contact mode at a scan rate of 0.5 Hz using EX-T125 probe with nominal resonance frequency 200–400 kHz and spring constant 13–77 Nm^–1^ (AnasysInstruments, United States).

### Dry Mass Measurement of Extracellular Vesicles Using Resonant Mass Measurement

The buoyant mass of the particles was measured with the Archimedes Particle Metrology System (Malvern Panalytical, Malvern, United Kingdom). The microchannel sensor used consisted of a microfluidic channel with a cross section of 2 × 2 μm^2^. In order to determine the dry mass and size of the EVs from the measured buoyant mass, EVs of known size were used to determine EV density. Monodisperse control EVs with 200 nm diameter were measured, and a spherical model was applied, from which a density of 1.4 g/cm^3^ was determined (Supplementary Figure 1). The sensitivity factor of the microchannel resonator was determined using monomodal gold calibration particles (NIST RM 8016, 60 nm, United States). From this sensitivity factor, the mass resolution of the sensor is ∼10^–15^ g, which for EVs corresponds to a limit of detection (LOD) of 100 nm.

### Quantification of Extracellular Vesicle Surface Markers Using nFCM

The monoclonal antibodies, anti-CD9 Alexa Fluor 488-conjugated (R & D systems, Canada), anti-CD63 Alexa Fluor 488-conjugated (Invitrogen, ThermoFisher Scientific), or anti-CD81 Alexa Fluor 488-conjugated (R & D systems, Canada) were used to assess protein surface markers of EVs. Approximately 1 × 10^10^ EVs/ml was stained using 8 μg/ml of each antibody and incubated 30 min at 37°C in dark condition. The EV samples were then washed with 2 ml of RNase-free PBS three times using ultracentrifugation 100,000 × *g*, 4°C, 70 min each. The supernatants were carefully aspirated from the bottom of the tubes in every wash. Subsequently, the pellets were dissolved in RNase-free PBS, and the fluorescence intensity was measured using a nFCM. The control sample was the basal medium with 0.05% (w/v) BSA without EVs. All fluorescence events were detected in FITC triggering of nFCM, and the threshold level was set by default in NF Profession 1.0 acquisition software.

### Quantification of Nucleic Acid Using nFCM

EVs isolated using TFF and ultracentrifugation were stained using 10 μM SYTO RNASelect green fluorescent cell stain (Invitrogen^TM^, ThermoFisher Scientific) at 37°C for 30 min. The EV samples were loaded on the exosome spin column MW 3000 (Invitrogen^TM^, ThermoFisher Scientific) to remove unbound dyes and measured using nFCM. The control sample was basal medium without EVs. All fluorescence events were collected for 120 s and fluorescence (FITC) triggering of an nFCM was used to detect fluorescence EVs. Data were analyzed using FlowJo software (version 10.6). The threshold level was set above the background level by the NF Profession 1.0 acquisition software by default.

### Quantification of Lipid Content Using nFCM

PKH67 and Diluent C (ThermoFisher Scientific) was selected to be the general membrane labeling for EVs. PKH67 was diluted in 100 μl of Diluent C to a final concentration of 15 μM (dye solution). Approximately 1 × 10^10^ EVs/ml were diluted with 80 μl of Diluent C, added to a dye solution, and incubated for 3 min with gentle pipetting. Excess dye was bound with 10% (w/v) BSA in RNase-free water. The EV samples were then diluted to 2 ml with RNase-free PBS and washed three times using ultracentrifugation 100,000 × *g*, 4°C, 70 min each. The pellet was gently resuspended in 100 μl of RNase-free PBS, and nFCM was used to measure the fluorescence intensity of EVs. The control sample was the basal medium only and the basal medium with 0.05% (w/v) BSA without EVs. All fluorescence events were triggered in the FITC channel and collected for 120 s. The threshold was set by default in NF Profession 1.0 acquisition software.

### Molecular Composition Analysis of Extracellular Vesicles Using Atomic Force Nanoscale Infrared Spectroscopy (AFM-IR)

The protocol for EV characterization using AFM-IR (nanoIR, Anasys Instruments, United States) was described in our previous study (Khanal et al., 2016). Briefly, each EV sample was placed on a zinc selenide prism and dried overnight. The laser signal was optimized before acquiring the nanoIR spectra ranging from 1,000 to 1,800 cm^–1^ at 4-cm^–1^ intervals with a scan rate of 0.5 Hz. A gold-coated tip and a silicon nitride cantilever with a nominal spring constant of 0.5 Nm^–1^ were used for all measurements. The acquired scan sizes were 10 × 5 μm for each sample, and Analysis Studio^TM^ software was used for data analyses. The “Savitzky–Golay” function was used to achieve smoothing of the spectra with the polynomial function of 3, and eight numbers of points.

### Measurement of Predicted Sedimentation of Extracellular Vesicles—Distorted Grid Model

The predicted transport modeling of TFF and ultracentrifugation isolated EVs, which was originally applied for the engineered nanomaterials (ENMs), was modified and adapted for EVs (Cohen et al., 2014). Briefly, the protocol comprised three interconnected parts: ENM dispersion preparation and characterization in suspension, effective density calculation, and delivered dose computation (Cohen et al., 2014). The dispersion preparation and characterization were not applied to EVs. The delivered dose metric was used to calculate the fraction of administered EVs in a 96-well plate over 24 h. Data were acquired using Matlab.

The effective density of EVs (ρ_*effective  density*_) was determined using the equation:

(1) $$\rho_{effectivedensity}=\rho_{media}+\left[\left(\frac{M_{EV}-M_{EVsol}}{V_{pellet}SF}\right)\left(1-\frac{\rho_{media}}{\rho_{EV}}\right)\right]$$

whereby:

ρ_*media*_ is the density of the medium (g/cm^3^).

*M*_*EV*_ is the total mass of EVs (g) in the dispensed volume of suspension.

*M*_*EVsol*_ is the mass of dissolved EVs in the dispensed volume of suspension.

*V*_*pellet*_ is the measured pellet size in centimeters squared inside the PCV tube.

*SF* is the stacking factor, which is the portion of the pellet that is composed of agglomerates (theoretical maximum of 0.74 for ordered stacking).

ρ_*EV*_ is the density of EVs (g/cm^3^).

### Visualization of Extracellular Vesicle Uptake Using Holotomography and Fluorescence Microscopy

CMSC29 EVs isolated using TFF and ultracentrifugation were labeled with PKH67 followed the lipid staining protocol as described in section ‘Quantification of Lipid Content Using nFCM’. The staining of EVs was done 1 h prior to incubation. Approximately 2 × 10^4^ BEAS-2B cells were dosed with 1 × 10^9^ PKH67-stained EVs/ml and incubated with the dye for 3 h. Uptake of EVs by cells was performed on a holotomography microscope Tomocube HT-2H (Tomocube Inc., Daejeon, South Korea). A water immersion objective (60×, N.A = 1.2) was used to acquire the images. Z-stacked images were acquired across each field-of-view, with a minimum of four field-of-views imaged for each type of EVs. The images were acquired in TomoStudio^TM^ 2.0 software, and they were further analyzed using ImageJ FIJI.

### Analysis of Cell Migration to Extracellular Vesicle Treatment After Lipopolysaccharides Injury

Cell migration in the presence of isolated EVs was measured by comparing the area of closure of a two-dimensional scratch wound. BEAS-2B cells were seeded at 1 × 10^4^ cells per well on Image Lock 96-well plates and allowed to adhere overnight. “Injury” was induced using 10 μg/ml of lipopolysaccharides (LPS) for 24 h (Xu and Zhou, 2020). A wound on the midline of culture well was then created using a 96-pin wound making tool (IncuCyte WoundMaker^TM^). After washing the cells with RNase-free PBS once, EVs isolated using TFF and ultracentrifugation were added at an ascending concentration ranging from 10 to 1,000 EVs per cell. Wound images were taken every 2 h with a 10× magnification objective lens using the IncuCyte live cell imaging system and IncuCyte ZOOM software program (Essen BioScience, United States). Wound confluence (%), which was represented as the wound closure (%), was assessed for all images using IncuCyte ZOOM software. Data were analyzed using GraphPad, and measurements of eight samples (*n* = 8) were performed for each condition.

### Analysis of Cellular Responses to Extracellular Vesicle Treatment After Lipopolysaccharide Injury

Cellular responses post-injury in the presence of TFF and ultracentrifugation isolated EVs were assessed by measuring intracellular nitric oxide (NO) levels. Approximately 5 × 10^3^ BEAS-2B cells were seeded on a glass bottom dish precoated in L-poline (MatTek) and allowed to adhere overnight. Cells were then exposed to 10 μg/ml of LPS for 24 h. Cells were next incubated with 50 μM NpNO1 probe for 24 h at 37°C, at 5% CO_2_. Excess NpNO1 probe was washed and imaged in FluoroBrite^TM^ DMEM media (Gibco, ThermoFisher Scientific) supplemented with 10% FBS and antibiotics (Pen/Strep). Samples were imaged using Olympus FV3000 microscope equipped with a 405-nm laser, a water 60× objective lens, and an incubator stage maintained at 37°C and 5% CO_2_. A minimum of three field-of-views were imaged for each condition per experiment with Z-stacked images per field-of-view. Maximum projected micrographs of the Z-stacks were presented in this study. Regions of interest were drawn around each cell, and mean fluorescence intensities were quantified using ImageJ FIJI. Statistical analyses were performed on the mean fluorescence intensity values and plotted using GraphPad.

### Statistical Analyses

Data were analyzed and presented as mean ± standard deviation (SD). For cell migration and cellular stress assays, a minimum of three independent preparations of each sample were made for each of the experiments (*n* = 3). Ordinary one-way ANOVA followed by Dunn’s multiple comparison’s test for pair-wise comparisons were used to determine the differences between multiple groups. A *P*-value <0.05 is considered to be statistically significant.

## Results

To measure EV size and concentration, we used particle tracking analysis (PTA), dynamic light scattering (DLS) (size only), nano-flow cytometry (nFCM), tunable resistive pulse sensing (TRPS), and asymmetric flow-field fractionation (AF4) (size only). Nanoscale infrared spectroscopy (AFM-IR) and nFCM were used to determine EV composition at the single EV and EV sub-population levels. For the first time, we used resonant mass measurement (RMM) for the characterization of dry mass and buoyant mass of large EVs (>100 nm) and distorted grid (DG) for the sedimentation prediction of EVs. Sedimentation of nanoparticles reveals the actual concentration of nanoparticles on the cell surface, which correlates with cellular uptake (Momen-Heravi et al., 2013). Therefore, sedimentation is the key factor in the interpretation of downstream biological effects of EVs on the cellular response. The actual functional effects of EV isolates on cells were determined using newly developed nitric oxide fluorescent probe to measure intracellular stress in an *in vitro* model of acute lung injury.

### Comparison of Extracellular Vesicle Size Distribution and Concentration

Chorionic and decidual MSC cell lines (CMSC29 and DMSC23) were used as cell sources to isolate EVs. EVs isolated from CMSC29 and EVs isolated from DMSC23 cells were referred to as CEVs and DEVs, respectively.

The size distribution of CEVs and DEVs was assessed using four independent techniques: PTA, DLS, nFCM, and TRPS.

### Particle Tracking Analysis

**FIGURE 1:**
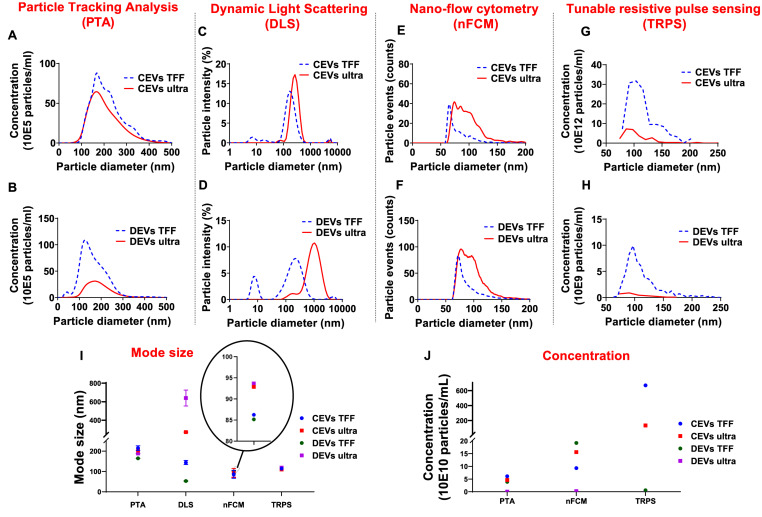
Comparison of the size distribution for different isolated extracellular vesicles (EVs). Representative histograms revealing size distribution of extracellular vesicles derived from chorionic mesenchymal stromal/stem cell (CEVs) and extracellular vesicles derived from decidual mesenchymal stromal/stem cell (DEVs) isolated using tangential flow filtration (TFF) and ultracentrifugation by four different methods. The size distributions of these EVs were assessed by **(A,B)** particle tracking analysis (PTA), **(C,D)** dynamic light scattering (DLS), **(E,F)** nano-flow cytometry (nFCM), and **(G,H)** tunable resistive pulse sensing (TRPS), respectively. The mode size **(I)** and concentration **(J)** of all EV samples were presented. The graphs represent the averaged values based on three repeated measurements of each EV sample.

PTA analyses showed that both CEVs and DEVs isolated using TFF and ultracentrifugation had a similar particle size distribution ranging from 100 to 300 nm (Figures 1A,B). However, there was a small peak at around 50 nm in the size distribution spectrum of DEVs isolated using TFF, which was not present in EVs isolated using ultracentrifugation (Figure 1B).

### Dynamic Light Scattering

Size analyses using DLS showed that the intensity-based size distribution of CEVs and DEVs, isolated using TFF and ultracentrifugation, were different (Figures 1C,D). CEVs isolated using TFF had one high-intensity peak at around 200 nm with one low-intensity peak at approximately 8 nm. In contrast, CEVs isolated using ultracentrifugation had one dominant peak at around 300 nm (Figure 1C). The intensity-based size distribution for DEVs isolated using TFF had two peaks at 8 and 200 nm, while DEVs isolated using ultracentrifugation had one broad peak at around 1,000 nm with one small shoulder at around 200 nm (Figure 1D).

The presence of small particles (around 8 nm) in CEVs isolated using TFF was verified using preliminary asymmetric flow-field fractionation (AF4) measurement (Supplementary Figure 2). Since the elution peak of CEVs isolated using TFF at 18 min coincides with pure BSA, the small-size particles in CEVs isolated using TFF were identified as BSA contaminants.

Overall, the size of both CEVs and DEVs isolated using ultracentrifugation as assessed by DLS were larger than the EVs isolated using TFF. This is likely to be related to the presence of a few larger entities (potentially agglomerates during the ultracentrifugation process). Since DLS is extremely sensitive to the presence of large entities, even with a low amount of these would account for the shape of the intensity-weighted data (Bishop et al., 1991).

### Nano-Flow Cytometry

nFCM measurements showed a broad size distribution ranging from 50 to 200 nm for both CEVs and DEVs isolated using TFF and ultracentrifugation (Figures 1E,F). While the size of most of EVs isolated using TFF (for both CEVs and DEVs) was around 60 nm, the size of EVs isolated using ultracentrifugation was evenly distributed from 50 to 200 nm.

### Tunable Resistive Pulse Sensing

TRPS measurements showed that CEVs isolated using TFF and ultracentrifugation had a similar size distribution spectrum (Figure 1G). The size of DEVs isolated using TFF showed a broad distribution ranging from 70 to 200 nm, while the size distribution obtained for DEVs isolated using ultracentrifugation could not be used for comparison due to a much lower sample concentration (Figure 1H).

The mode sizes of both CEVs and DEVs were consistent for each individual method (with differences between methods) regardless of the isolation method. PTA, nFCM, and TRPS showed that the mode sizes of CEVs and DEVs were around 150, 90, and 110 nm, respectively (Figure 1I). However, DLS showed differences in mode sizes of CEVs and DEVs depending on the isolation method. Ultracentrifugation yielded larger CEVs with mode size around 270 nm, while CEVs isolated with TFF was approximately 197 nm. Similarly, the mode size of DEVs isolated using ultracentrifugation was approximately 1,120 nm, and TFF isolated DEVs was approximately 240 nm. It is important to notice that the scattering intensity depends on the 6th power of the size of the macromolecules, therefore, large agglomerates—even a very small amount will overwhelm the intensity from small size particles in DLS (Barnett, 1942). Thus, the observation of small size particles in EVs isolated using TFF in DLS indicated the substantial amount of small size particles and the absence of agglomerates in TFF isolated EVs. To further explore the possibility of the presence of small size particles in the samples, AF4 was used (Supplementary Figure 2) to fractionate and measure the hydrodynamic diameter of the entities as they eluted.

The concentration of EVs was determined using three techniques: PTA, nFCM, and TRPS (Figure 1J). The concentration of CEVs isolated using TFF and ultracentrifugation determined by PTA and nFCM was approximately 10^11^ EVs/ml. In contrast, TRPS measured approximately 10^12^ CEVs/ml isolated using TFF, one order of magnitude larger than PTA and nFCM. The concentration of DEVs isolated using TFF measured using PTA and TRPS was ∼10^9^ EVs/ml, while the measurement made using nFCM was 10^11^ EVs/ml, two orders of magnitude higher. The concentration of DEVs isolated using ultracentrifugation was consistent for all measurement methods, 10^9^ EVs/ml.

In summary, both PTA and nFCM measurements showed a consistent average size for both DEVs and CEVs regardless of isolation method; however, the concentration of EVs was around two orders of magnitude lower for DEVs isolated using ultracentrifugation. Overall, results suggested that PTA was less sensitive than nFCM for the detection of EVs smaller than 100 nm.

### Mass Analysis

Besides the size distribution, the buoyant mass and dry mass of different isolated EVs were quantified. Previously, buoyant mass and dry mass were used to determine the exact amount of nanoparticles interacting with cells and tissues in toxicity studies (Cohen et al., 2013). Here, we used resonant mass measurement (RMM) for the first time to characterize EVs for buoyant mass and dry mass. The advantage of this approach is that both mass parameters can be measured for individual vesicles, which in turn reveals the total mass, including molecular cargo, of each vesicle (Rupert et al., 2017). Precise knowledge of the amount of EV cargo is pivotal to define the biological function of EVs.

**FIGURE 2:**
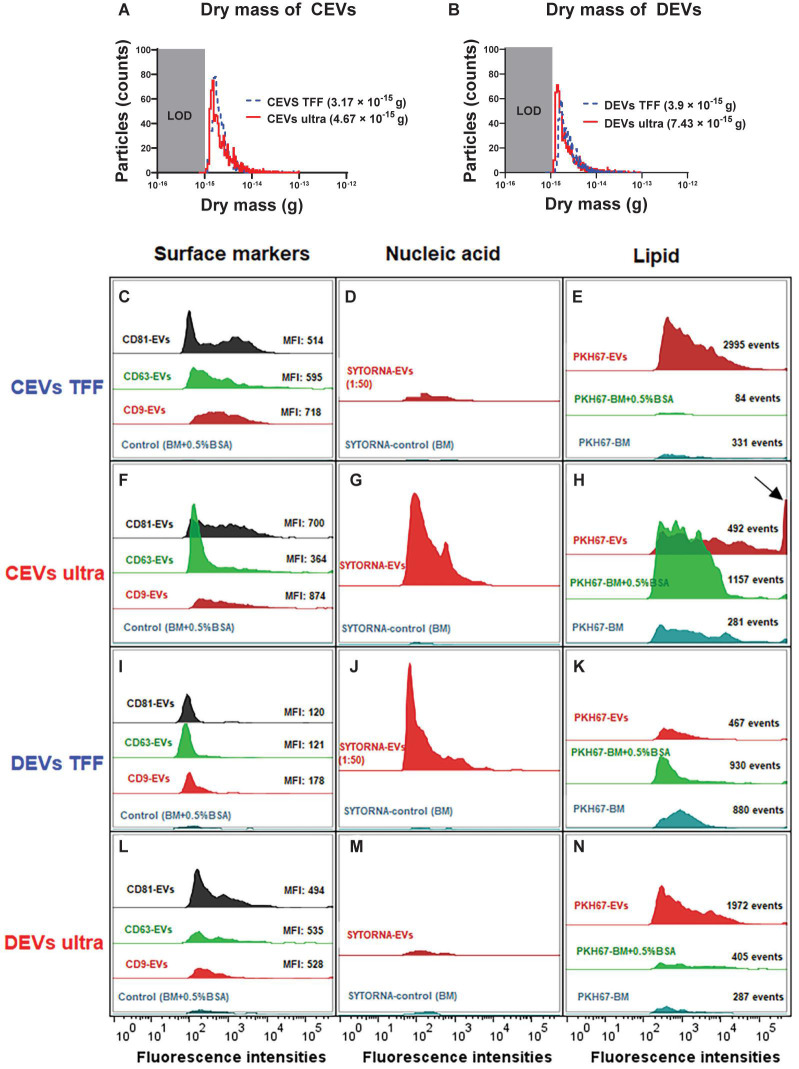
The dry mass of different isolated EVs, and EV molecular composition was analyzed using nano-flow cytometry (nFCM). The dry mass of TFF and ultracentrifugation-isolated **(A)** CEVs and **(B)** DEVs. LOD, limit of detection. Representative histograms showing the presence of three surface markers CD9, CD63, and CD81 **(C,F,I,L)**, nucleic acid **(D,G,J,M)**, and lipid content **(E,H,K,N)** in CEVs and DEVs isolated using TFF and ultracentrifugation. The arrow indicates the saturated fluorescence peak. MFI, mean fluorescence intensity.

The dry mass of EVs isolated using ultracentrifugation and TFF was measured and presented in Figures 2A,B. The size distribution of each EV type was calculated from the measured buoyant mass, with a particle density of 1.4 g/cm^3^ (Supplementary Figure 3). It is important to notice that the limit of detection (LOD) of RMM for dry mass is 10^–15^ g, and for size distribution, it is 100 nm. Therefore, only sub-population of EVs with the high mass was detected and measured. When plotted as a function of dry mass, both detectable CEVs and DEVs isolated using ultracentrifugation showed higher dry mass than EVs isolated using TFF (Figure 2). The dry mass of measurable CEVs isolated using ultracentrifugation (4.67 × 10^–15^ g) was higher than CEVs isolated using TFF (3.17 × 10^–15^ g) (Figure 2A). Similarly, measurable DEVs isolated using ultracentrifugation (7.43 × 10^–15^ g) showed higher dry mass than DEVs isolated using TFF (3.9 × 10^–15^ g) (Figure 2B). Interestingly, a sub-population of DEVs isolated using TFF, which has a positive buoyancy was detected, and its size was estimated using a density of 0.01 g/cm^3^ (Supplementary Figure 3). This positively buoyant sub-population of DEVs isolated using TFF could be bubbles or empty vesicles. The higher mass for detectable EVs (above 100 nm) (CEVs and DEVs) isolated using ultracentrifugation suggested the presence of more agglomerates in ultracentrifugation-isolated EVs.

Cumulatively, size and mass measurements suggested that these EVs contained some amounts of agglomerates. The agglomeration of EVs during ultracentrifugation was reported in previous studies (Linares et al., 2015; Nordin et al., 2015; Taylor and Shah, 2015).

### Extracellular Vesicle Molecular Composition Assessed Using Nano-Flow Cytometry and Nanoscale Infrared Spectroscopy

#### Extracellular Vesicle Surface Markers

The differences in the dry mass of EVs isolated using ultracentrifugation, and TFF suggested that there were differences in molecular composition of EVs. Therefore, we assessed the presence of three common EV surface markers (i.e., CD9, CD63, and CD81) on different isolated EVs by using nFCM. CD9, CD63, and CD81-positive were EVs defined as the events that were above the threshold level and detected by fluorescence triggering (Supplementary Figure 4). A control using basal medium with 0.5% (w/v) BSA (Supplementary Figure 4) showed negligible fluorescence in fluorescence triggering, which demonstrated that the free dye was successfully removed by our washing procedure. The histogram analyses showed that both CEVs and DEVs regardless of isolation method were positive for CD9, CD63, and CD81 (Figures 2C,F,I,L). The overall mean fluorescence intensity (MFI) of CD9, CD63, and CD81 was higher for CEVs isolated using the same method. Profile analysis of individual surface markers showed that their expression was not uniform across different sub-populations of EVs. CD9 was detected as strongly positive in CEVs isolated using TFF (MFI: 718), followed by CD63 (MFI: 595) and CD81 (MFI: 514) (Figure 2C), whereas the expression of CD9 (MFI: 874) and CD81 (MFI: 700) was higher than CD63 (MFI: 364) in CEVs isolated using ultracentrifugation (Figure 2F).

The expression of the markers was more uniform for DEVs than CEVs when using the same isolation method. All three markers of DEVs isolated using TFF were uniformly detected at a low MFI level (under 200) (Figure 2I). Specifically, the MFI value for CD9 (178) was higher than CD63 and CD81 (121 and 120, respectively). Since DEVs isolated using TFF had a substantial amount of small size particles (∼8 and 200 nm) assessed using DLS, the expressions of the markers were lowest. Meanwhile, DEVs isolated using ultracentrifugation showed a higher expression of the markers than TFF. The MFI values for CD9, CD63, and CD81 were 494, 535, and 528, respectively, in DEVs isolated using ultracentrifugation (Figure 2L).

The profile of the surface markers for EVs isolated from each of the cell type was different depending on the isolation method. This result suggested that each of the isolation method provided different EV populations. However, regardless of the isolation method, EVs isolated from DMSC23 consistently showed more uniform expression of all three markers, which implied that exosome-specific markers were more homogenously expressed on DEVs.

#### Nucleic Acid Profiling

We next quantify the total nucleic acid content inside EVs by staining EVs with SYTO RNASelect green fluorescent cell stain. The percentage of nucleic acid was calculated by dividing the concentration of SYTO RNASelect green-positive events by the total concentration of EVs (Table 1). EVs isolated using TFF were diluted 50 times before staining to achieve the same threshold level as EVs isolated using ultracentrifugation. Since TFF isolated EVs were diluted, a smaller fluorescence intensity peak was shown in CEVs isolated using TFF in comparison with CEVs isolated using ultracentrifugation (Figures 2D,G). However, the analyses after the calculation showed that CEVs isolated using TFF contained 22 times more of the nucleic acid (2.46%) than CEVs isolated using ultracentrifugation (0.11%) (Table 1). Similarly, the nucleic acid content of DEVs isolated using TFF (3.31%) was seven times higher than DEVs isolated using ultracentrifugation (0.46%) (Table 1 and Figures 2J,M). Overall, we concluded that the total amount of nucleic acid in DEVs was higher than CEVs when using the same isolation method. The low percentage of SYTO RNASelect green-positive events could be due to the low amount of RNA inside EVs. Moreover, since the nucleic acids are considered to be small molecules and may have low fluorescence intensities, the fluorescence events from the nucleic acid may fall below the detection limit of nFCM.

**TABLE 1:** The percentage of nucleic acid content in extracellular vesicles derived from chorionic mesenchymal stromal/stem cell (CEVs) and extracellular vesicles derived from decidual mesenchymal stromal/stem cell (DEVs) isolated using tangential flow filtration (TFF) and ultracentrifugation assessed using nano-flow cytometry (nFCM).

EV type	Percentage of nucleic acid (%)
CEVs TFF	2.46
CEVs ultra	0.11
DEVs TFF	3.31
DEVs ultra	0.46

#### Lipid Membrane Profiling

We next assessed the lipid content in each EV type by staining EVs with green fluorescence lipophilic dye, PKH67. PKH67, which labels the cell membrane by inserting its aliphatic chains into the lipid membrane, has been used extensively to label the lipid membrane of EVs (Ohno et al., 2013). The lipid compositions of DEVs and CEVs were measured and compared with two controls: basal medium only (BM) and basal medium with 0.5% (w/v) BSA (BM + 0.5% BSA) (Figure 2). We used two controls as PKH67 may label other components in the medium (non-specific binding) (Lai et al., 2015; Takov et al., 2017). Hence, two control groups were essential to eliminate false positives due to fluorescence from non-EV components.

The total fluorescence events of CEVs isolated using TFF (2,995 events) was nine- and 35-fold higher than BM (84 events) and BM + 0.5% BSA (331 events) (Figure 2E), which showed substantial PKH67-positive EVs. On the other hand, CEVs isolated using ultracentrifugation (492 events) had 221 more fluorescence events than BM (281 events), but they had 665 less events than BM + 0.5% BSA (1,157 events) (Figure 2H). The fluorescence-positive events in controls indicated that there were some “contaminants” in the controls, which bound to PKH67 and caused the fluorescence. The saturated fluorescence intensity peak in CEVs isolated using ultracentrifugation, which was not detected in controls, suggested the presence of larger size vesicles or agglomerates (Figure 2H; arrow).

The total fluorescence events of DEVs isolated using TFF (467 events) were approximately twofold lower than BM (880 events) and BM + 0.5% BSA (930 events) (Figure 2K). DEVs isolated using ultracentrifugation (1,972 events) had 1,685 and 1,567 more events than BM (287 events) and BM + 0.5% BSA (405 events), respectively (Figure 2N). This result indicated that DEVs isolated using ultracentrifugation contained a high concentration of PKH67-positive EVs. Since DEVs isolated using ultracentrifugation had the lowest concentration of EVs among all EV groups, this result could be a false-positive result. One possible explanation could be that during ultracentrifugation, lipid–protein aggregates and other media components are isolated beside EVs. These undesired components can potentially bind PKH67, which causes a misleading result.

As observed in Figures 2H,N, the number of events shown in the controls (BM and BM + 0.5% BSA) emphasized the non-selective binding of PKH67 to other components. These results are consistent with the findings that the PKH67 lipophilic dyes is not specific to EVs (Lai et al., 2015). Therefore, this study was inconclusive regarding to the amount of lipid of EV isolates.

### Molecular Composition of Individual Extracellular Vesicles Using Nanoscale Infrared Spectroscopy

**FIGURE 3:**
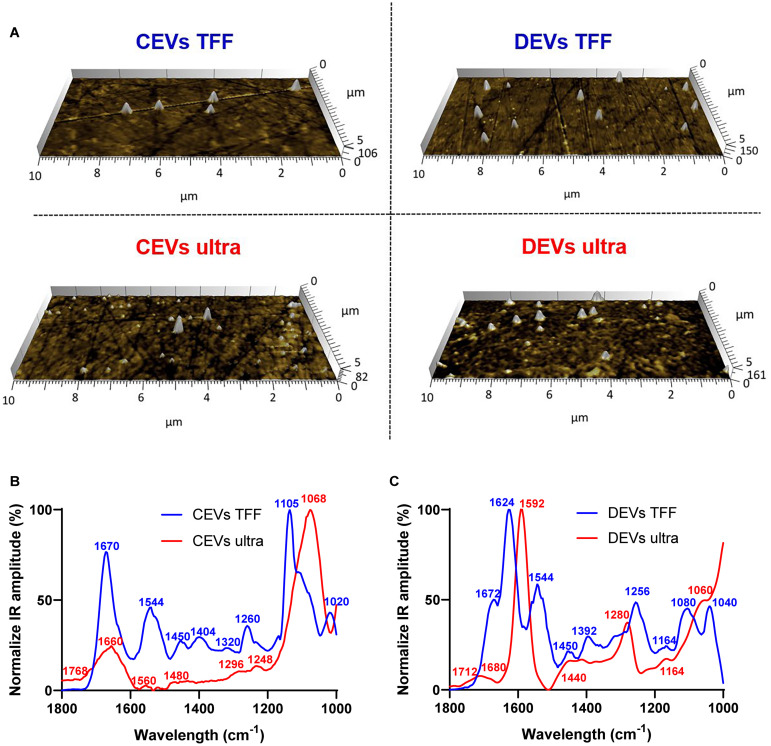
Average infrared absorption spectra of extracellular vesicle (EV) molecular composition corresponding topographical atomic force microscopy (AFM) images for EVs. **(A)** AFM images (10 × 5 μm) of individual EVs (indicated as white protrusions) deposited on prism. Normalization of the average AFM-IR spectra of **(B)** CEVs isolated using TFF and ultracentrifugation; **(C)** DEVs isolated using TFF and ultracentrifugation. At least 15 individual EVs were randomly selected and analyzed using Analysis Studio^TM^ software.

While nFCM proved to be a very effective method to determine size and composition of EVs at bulk population level, this method, similar to other size measurements methods, cannot effectively distinguish between a single large EV and an agglomerate of several small EVs (Linares et al., 2015). Therefore, to characterize EVs at single vesicle level, we used nanoscale infrared spectroscopy (AFM-IR) (Figure 3; Kim et al., 2018, 2019b). Topographical AFM images showed that both CEVs and DEVs were spherical and their size ranged between 20 and 300 nm regardless of isolation method (Figure 3A). Molecular analysis at the single vesicle level employed state-of-the-art nanoscale infrared spectroscopy (AFM-IR), which showed that individual EVs contained proteins, nucleic acids, and lipids (Supplementary Figure 5 and Figures 3B,C). However, the compositions of both types of EVs were different depending on the isolation method.

The spectra of CEVs isolated using TFF had dominant peaks for all three main components of EVs: protein (1,670, 1,544, and 1,260 cm^–1^), nucleic acid (1,450, 1,404, and 1,320 cm^–1^), and lipid (1,105 and 1,020 cm^–1^), which confirmed the presence of all three components in individual EVs (Lee et al., 2017). The intensity ratio of protein amide I (1,670 cm^–1^) and amide II (1,544 cm^–1^) was 2:1, which indicated ordinary protein conformation in CEVs isolated using TFF. Peaks at 1,450 and 1,404 cm^–1^ in the spectra of CEVs isolated using TFF were attributed to phosphatidylcholine head group and thymine of RNAs (Kim et al., 2019b). In contrast, the spectra of CEVs isolated using ultracentrifugation had a broad band with low intensity in amide I peak at 1,660 cm^–1^. They also showed a dominant peak at 1,068 cm^–1^ (lipid) and five minor peaks at 1,768, 1,560, 1,480, 1,296, and 1,248 cm^–1^. Since the intensity of amide II peak (1,560 cm^–1^) was low in the spectra of CEVs isolated using ultracentrifugation, it suggested that protein conformation for these EVs was altered. Moreover, the absence of peaks in the 1,450–1,350 cm^–1^ region in the spectra of CEVs isolated using ultracentrifugation suggested smaller amounts of nucleic acid (i.e., RNAs), which was consistent with the nFCM result (Table 1). The dominant peak at ∼1,068 cm^–1^ in the spectra of CEVs isolated using ultracentrifugation was associated with the ester C–O–C symmetric stretching vibration (Mohan et al., 2020). This peak was shifted to ∼1,105 cm^–1^ in the spectra of CEVs isolated using TFF. The peak at 1,768 cm^–1^ in the spectra of CEVs isolated using ultracentrifugation could be related to ester groups, primarily from lipid and fatty acids (Kim et al., 2018).

The spectra of DEVs isolated using TFF characterized showed dominant peaks at 1,624, 1,544, 1,256 cm^–1^, and a shoulder at 1,672 cm^–1^ (protein), 1,450, 1,392 cm^–1^ (nucleic acid), and 1,080 and 1,040 cm^–1^ (lipid). The intensity ratio of amide I (1,624 cm^–1^) and amide II (1,544 cm^–1^) peaks was ∼2:1, which suggested unaltered protein conformation in the spectra of DEVs isolated using TFF. Peaks at 1,450 and 1,392 cm^–1^ in the spectra of DEVs isolated using TFF confirmed the presence of RNAs (Kim et al., 2019b). In contrast, the spectra of DEVs isolated using ultracentrifugation had dominating peaks at 1,592 cm^–1^ (protein amide II), 1,280 cm^–1^ (nucleic acid, protein), and broad bands at 1,680, 1,440, and 1,164 cm^–1^. The low intensity of the amide I (1,680 cm^–1^) peak in the spectra of DEVs isolated using ultracentrifugation suggested some alterations in protein structures of these EVs. The broad band with low intensity at 1,440 cm^–1^ in the spectra of DEVs isolated using ultracentrifugation suggested a reduced amount of RNAs, which was consistent with the nFCM result (Table 1). In addition, the bands in the 1,080–1,040 cm^–1^ region were associated with vibration from phosphate stretch of RNA/DNA or lipid (Balan et al., 2019). While the spectra of DEVs isolated using TFF had two peaks at 1,080 and 1,040 cm^–1^, the spectra of DEVs isolated using ultracentrifugation only had one peak at 1,060 cm^–1^ suggesting changes in RNA/DNA structures and lipid. The peak at 1,712 cm^–1^ in the spectra of DEVs isolated using ultracentrifugation could be related to ester groups, primarily from lipid and fatty acids.

Overall, the structures of all three key molecular/structural components of EVs, proteins, lipids, and nucleic acid were maintained in the spectra of both CEVs and DEVs isolated using TFF. In contrast, for the spectra of EVs isolated using ultracentrifugation, the intensity, position, and the width of the peaks changed, indicating changes to the molecular structure of these components. These changes were observed for both CEVs and DEVs.

Since both nucleic acid and lipid peaks are centered around the same frequency, it was necessary to validate the results using an alternative technique, e.g., nFCM. We showed that the amount of nucleic acid in the spectra of EVs isolated using ultracentrifugation was smaller than TFF regardless of the origin of EVs, which was consistent with the nFCM result. Therefore, combining both nFCM and nanoscale infrared spectroscopy are necessary to gain precise understanding of the molecular/structural composition of individual EVs.

### Predicted Sedimentation of Isolated Extracellular Vesicles Using Distorted Grid Model

The differences in physicochemical properties of EV types suggested that their colloidal stability, interactions with the cell membrane, internalization, and downstream biological effects could also be different (Willms et al., 2018). While largely ignored, the colloidal stability is a critical parameter that shows ability of EVs to move toward, and reach cells (“sediment”), hence, likely to affect the biological effectiveness of EVs. The transport modeling for EVs was completed for the first time based on their size, size distribution, effective density to calculate the particles sedimentation, and diffusion in cell culture media.

**FIGURE 4:**
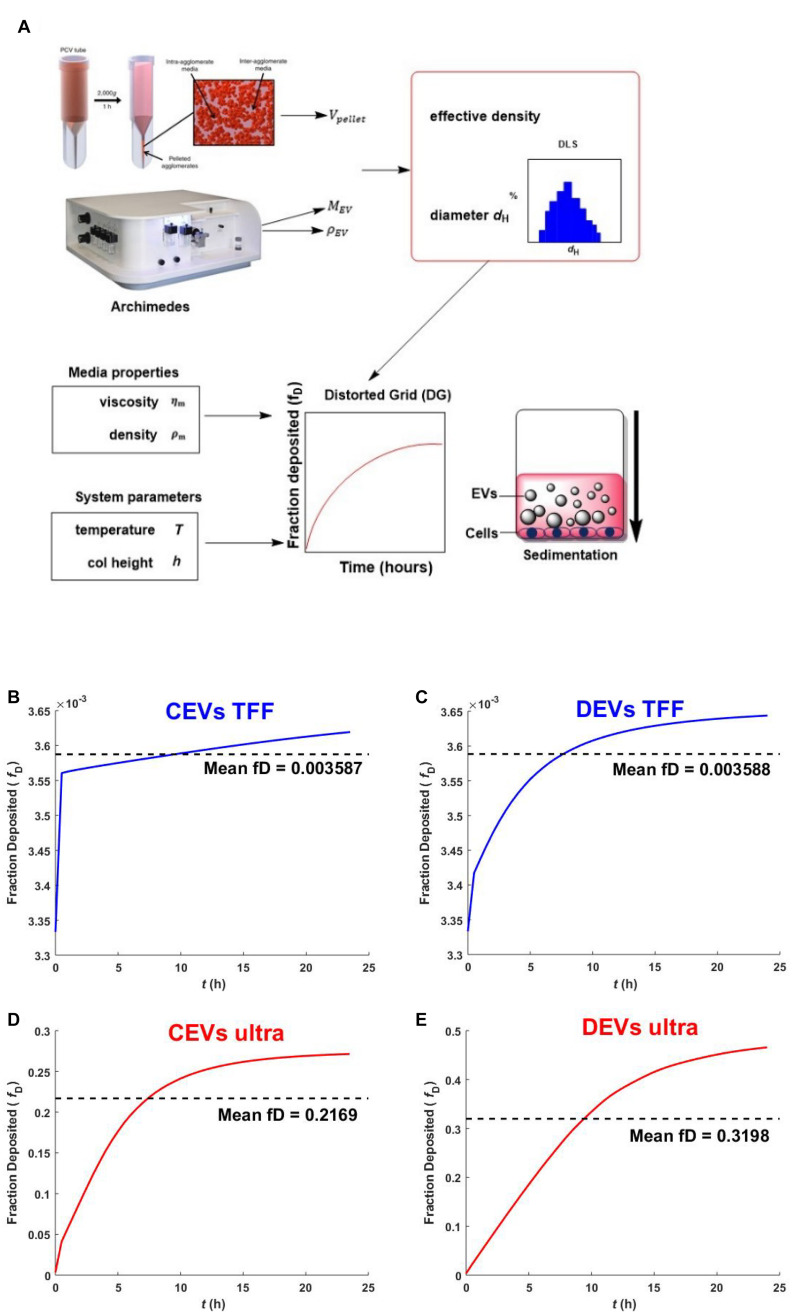
Schematic overview of nanodosimetry approach and the predicted fraction deposited of EVs. The schematic overview of DG modeling adapted from DeLoid et al. (2017) **(A)**. The predicted fraction deposited of CEVs isolated using **(B)** TFF and **(D)** ultracentrifugation; DEVs isolated using **(C)** TFF and **(E)** ultracentrifugation into each well in a 96-well plate was generated by Matlab. Mean fD is the mean fraction deposited.

The predicted sedimentation of different EV isolates was assessed using the DG model. All the values used for the modeling are shown in Supplementary Table 2. The results indicated the differences in sedimentation between EVs depending on the isolation method (Figure 4). CEVs isolated using TFF showed that the deposited fraction was predicted to reach 0.00354 within 1 h, then slowly reach the mean fraction deposited (0.003587) after ∼10 h (Figure 4B). Meanwhile, CEVs isolated using ultracentrifugation was predicted to reach the mean fraction deposited (0.2169) after ∼7.5 h (Figure 4D). For DEVs, the mean fraction deposit was predicted to be reached after ∼8 h for EVs isolated using TFF (0.003588)(Figure 4C) and ∼10 h for EVs isolated using ultracentrifugation (0.3198)(Figure 4E). Overall, the mean of fraction deposit of EVs isolated using ultracentrifugation was 65 and 100 times higher than EVs isolated using TFF for CEVs and DEVs, respectively. A previous study reported that less agglomeration resulted in a smaller deposited fraction (DeLoid et al., 2015), which confirmed that EVs isolated using TFF do not contain agglomerates. In contrast, EVs isolated using ultracentrifugation may contain the agglomerates, and these agglomerates could be attributed to the higher sedimentation. This result was consistent with our size distribution (DLS), mass (RMM), and the visualization of EV (holotomography) internalization by a single cell (see below).

### Extracellular Vesicles Internalization by Living BEAS-2B Cells Using Holotomography

**FIGURE 5:**
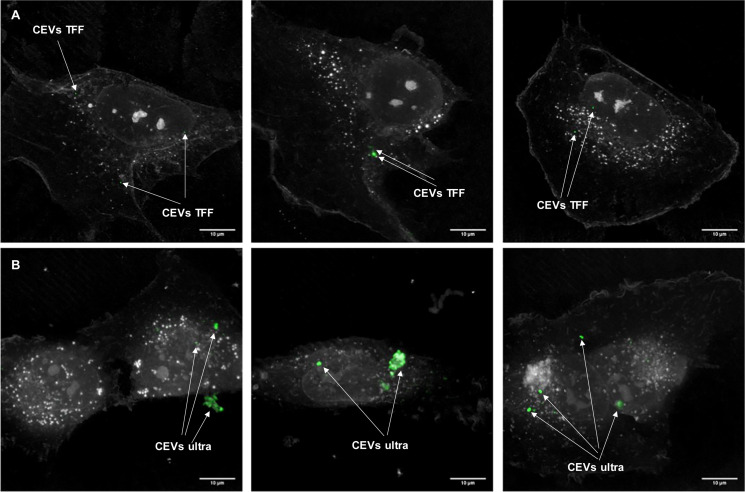
The localization of EVs with BEAS-2B cells. Three representative images of BEAS-2B cells and stained CEVs (green color) isolated using **(A)** TFF and **(B)** ultracentrifugation. Scale bar represents 10 μm. Bright white dots inside BEAS-2B cells indicate the lipid droplets.

To attest the relevance of the predicted sedimentation study in assessing EV uptake, we used EVs isolated using TFF and ultracentrifugation and visualized their presence in and on single cells using correlative holotomography and fluorescence microscopy (Figure 5). This method, unlike confocal microscopy, does not require any fluorescent labeling for cells, which imparted no stress on cells. Additionally, holotomography microscopy enables us to visualize the differences in refractive index; thus, we can observe subcellular organelles of the cells without labeling with nanometer resolution (Kim et al., 2020).

DEVs isolated using TFF had a substantial amount of small size particles (Figure 1), which caused the difficulties in visualizing EVs. Therefore, to demonstrate the differences between TFF and ultracentrifugation-isolated EVs, the EVs localization study using a holotomography microscope was conducted only for CEVs. BEAS-2B cells were dosed with PKH67-labeled CEVs and imaged 3 h later. We observed fluorescence inside the cells and not on the plasma membrane, which suggested that EVs were internalized by the cells. In order to confirm whether EVs are inside cell cytoplasm, we used digital staining for cells and processed them into dynamic 3D images based on the rotation of one axis (Supplementary Video 1). The analysis of the dynamic images evidenced that EVs were internalized by BEAS-2B cells after 3 h. The results showed that the punctate CEVs isolated using TFF were qualitatively and homogenously distributed in the cytoplasm (Figure 5A) and are of uniform sizes. The size of CEVs isolated using ultracentrifugation were heterogenous, ranging from 100 to 500 nm (Figure 5B). These observations confirmed agglomeration had occurred during ultracentrifugation. The control experiment using BM + 0.5% BSA showed no fluorescently labeled particles (data not shown).

### Cell Migration Assay With Different Concentrations of Isolated Extracellular Vesicles

**FIGURE 6:**
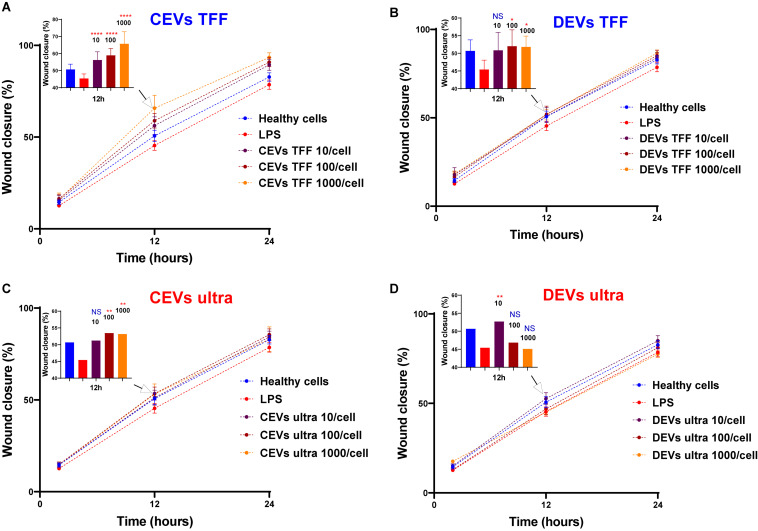
Assessment of wound closure in injured BEAS-2B in response to EV treatment. BEAS-2B cells were “injured” with 10 μg/ml of LPS and treated with CEVs isolated using **(A)** TFF and **(C)** ultracentrifugation; DEVs isolated using **(B)** TFF and **(D)** ultracentrifugation. Error bars represent standard deviation (SD), *n* = 8. Statistics significance was determined by ordinary one-way ANOVA test, followed by Dunn’s multiple comparisons test for pair-wise comparisons. Statistical significance shown was between LPS treatment and other groups at time point 12 h. ^∗^*P* < 0.1; ^∗∗^*P* < 0.01; ^****^P < 0.0001; NS, not significant.

The actual biological effects of different EV isolates were investigated using the scratch wound assay, which was modified to measure the wound closure of cells toward the “wound.” Lung epithelial cells (BEAS-2B) were selected, and 10 μg/ml of lipopolysaccharides (LPS) was used before wound scratch as an *in vitro* model of acute lung injury. LPS is a key pathogenic factor that induces various inflammatory mediators, which resulted in lung inflammatory and epithelial damage (Nova et al., 2019). Concentrations of CEVs and DEVs isolated using TFF and ultracentrifugation, ranging from 10 to 1,000 EVs per cell, were used in order to test their ability to promote cell migration and increase wound closure after injury. Both CEV- and DEV-treated cells enhanced wound closure percentage when compared with LPS-treated only cells (Figure 6). Increasing the concentration of CEVs isolated using both TFF and ultracentrifugation (from 10 to 1,000 EVs per cell) increased the wound closure percentage of BEAS-2B cells (Figures 6A,C). Especially, at the concentration of 10 EVs per cell for CEVs isolated using TFF, the wound closure percentage was significantly increased compared with LPS-treated only cells (Figure 6A).

Treatment with DEVs isolated using TFF resulted in increased wound closure percentage of BEAS-2B cells (Figure 6B). The wound closure percentage of BEAS-2B was significantly increased at the concentration of 100 and 1,000 EVs per cell for DEVs isolated using TFF. In contrast, with a high concentration of DEVs isolated using ultracentrifugation (100 and 1,000 EVs per cell), the wound closure percentage remains as low as the LPS-treated cells (Figure 6D). This was supported by the highest sedimented amount for DEVs isolated using ultracentrifugation using the DG model, which resulted in the highest dose for cells. We postulate that the high DEV dose could be causing adverse effects on the cells.

### Cellular Stress After Injury in Response to Different Concentration of Isolated Extracellular Vesicles

**FIGURE 7:**
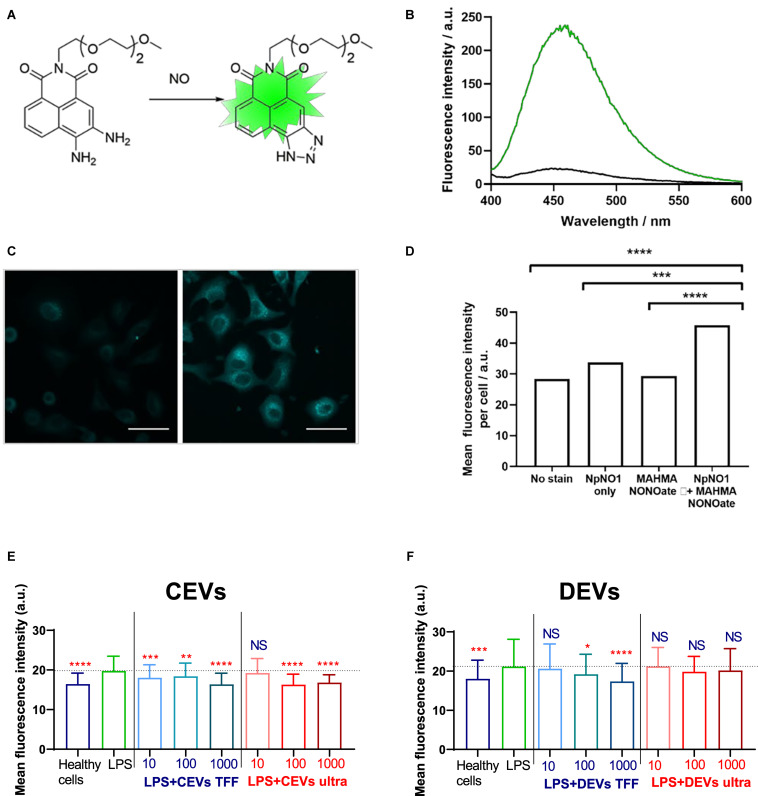
A novel, water soluble fluorescent sensor for intracellular nitric oxide (NO) and assessing NO levels in BEAS-2B cells. **(A)** Water-soluble NO sensor NpNO1 and the product of its reaction with nitric oxide to give a fluorescent triazole derivative. **(B)** Fluorescence emission response of NpNO1 (10 μM) in the presence of diethylamine NONOate (1 mM). **(C)** Representative micrographs of A549 cells treated with NpNO1 (50 μM) in the absence (left) and presence (right) of MAHMA NONOate (5 mM). Scale bar represents 50 μm. **(D)** Mean fluorescence intensity of A549 cells treated as indicated measured across at least three different field of views in each of the three independent experiments performed (*n* = 3). MAHMA NONOate, (Z)-1-{N-methyl-N-[6-(N-methylammoniohexyl)amino]}diazen- 1-ium-1,2-diolate, an NO donor. BEAS-2B cells were “injured” with 10 μg/ml of LPS and dosed with **(E)** CEVs isolated using TFF and ultracentrifugation and **(F)** DEVs isolated using TFF and ultracentrifugation. Statistical significance was determined by ordinary one-way ANOVA, followed by Dunn’s multiple comparisons test for pair-wise comparisons, *n* = 3. Statistical significance shown was between LPS treatment and other groups. ^∗^*P* < 0.1; ^∗∗^*P* < 0.01; ^∗∗∗^*P* < 0.001; ^****^P < 0.0001; NS, not significant. Error bars denote standard deviation (SD). The dash line represents the mean level of the LPS group.

Nitric oxide (NO) measurement allows the measurement of cellular stress levels in response to injury (Nasyrova et al., 2015). NO participates in diverse physiological and pathological processes, such as inflammation (van der Vliet et al., 2000). In response to inflammatory stimuli, NO production is markedly elevated (Coleman, 2001). Given the importance in studying and understanding NO for health and disease, a number of fluorescent sensors have been reported to date (Iverson et al., 2018). However, a common feature of small molecule sensors is poor water solubility, arising from the highly aromatic structures. This has two main drawbacks in cellular studies: first, a need to prepare a stock solution of the dye in an organic solvent such as dimethyl sulfoxide (DMSO), which itself can perturb the system, and second, a tendency for the dye to aggregate and self-quench. We therefore sought to prepare a water-soluble fluorescent sensor for NO by utilizing the previously-reported selective conversion of aromatic ortho-diamines to triazoles in the presence of nitric oxide and oxygen (Iverson et al., 2018), conjugating this reactive group to a 4-amino-1,8-naphthalimide fluorophore, which we have previously shown to have great potential for sensing applications (Leslie et al., 2018; Yang et al., 2018). Water solubility was achieved by incorporating a triethylene glycol (TEG) group to give the final probe, NpNO1 (Figure 7A). NpNO1 was prepared in four synthetic steps from commercially available bromoacetic naphthalic anhydride, in 42% overall yield as detailed in the Supplementary Information (Scheme 1). NpNO1 was found to have excellent aqueous solubility up to 1 mM (Supplementary Figure 6) and a strong fluorescence turn-on at 455 nm with NO addition (Figure 7B).

The ability of NpNO1 to detect changes in intracellular NO levels by confocal microscopy was confirmed in A549 (human alveolar basal epithelial) cells treated with 50 μM of NpNO1 overnight, in the presence or absence of 5 mM MAHMA NONOate (Figure 7C and Supplementary Figure 6). MAHMA NONOate spontaneously releases NO and, hereafter, will be referred to as the NO donor. We observed minimal/basal fluorescence from untreated cells (no stain), cells treated with only MAHMA NONOate, or cells treated with NpNO1 alone. In A549 cells treated with both the NpNO1 and the NO donor, we observed fluorescent puncta in every cell, and a significant increase in mean fluorescence intensity.

We then assessed intracellular NO levels after adding various EVs, by quantifying the NpNO1 fluorescence intensity in cells imaged using confocal microscopy (Supplementary Figure 7).

Similar to the scratch wound assay, we modeled cellular injury by treating human BEAS-2B epithelial cells with 10 μg/ml of LPS. The intracellular NO, as indicated by the fluorescence intensity of NpNO1, increases after LPS injury (Figure 7), which is consistent with the literature (Chokshi et al., 2008). Next, we assessed the fluorescence intensity of NpNO1, which corresponds to the levels of intracellular NO present, in LPS-treated cells dosed with various isolated EVs. Upon adding the concentration of 10–1,000 EVs/cells for CEVs isolated using TFF, the NO levels diminished compared with LPS-treated cells (Figure 7E), whereas, dosing the LPS-treated cells with 10 EVs/cell for CEVs isolated using ultracentrifugation, the intracellular NO levels remain high, as in the LPS-treated cells (Figure 7E). The intracellular NO levels decreased when 100 and 1,000 EVs/cells for CEVs isolated using ultracentrifugation were added compared with LPS-treated cells.

The NpNO1 fluorescence intensity remained as high as LPS-treated cells using the concentration of 10 DEVs/cell isolated using TFF (Figure 7F). The intracellular NO levels, as indicated by NpNO1 fluorescence intensity, decreased when the cells were treated with 100 and 1,000 DEVs isolated using TFF. For DEVs isolated using ultracentrifugation, the intracellular NO levels remain as high as the LPS-treated cells at all concentrations (Figure 7F).

## Discussion

While there is palpable excitement about the future of EV-based medicine, a current major limitation is the lack of understanding about how to effectively and uniformly isolate them from complex biological milieu (Li et al., 2019). Furthermore, it has not been clear whether different isolation methods extract different sub-populations of EVs, which would impact on their downstream applications. This gap in knowledge is associated with conceptual and technical limitations in EV characterization. We have addressed this challenge by using multiple techniques including optical, non-optical, and high-resolution single vesicle characterization methods, and we provide extensive experimental evidence that *isolation method determines the composition and biological function of EV isolates*.

Two common isolation methods were used in this study: ultracentrifugation and TFF. Using four different techniques, we demonstrated that the size distributions of EVs isolated using ultracentrifugation and TFF were different. Since EVs are heterogenous and each characterization technique has its own limits of detection (Gandham et al., 2020), it is essential to measure the size distribution using multiple techniques. Here we used four techniques to measure the size distribution and concentration of EVs including PTA, DLS, nFCM, and TRPS. PTA is based on light scattering and Brownian motion, which cannot differentiate between large protein aggregates and single large particles (Gercel-Taylor et al., 2012). For DLS, the size distribution depends on the intensity of light scattering; therefore, the intensity of the scattered light from small particles is normally surpassed by large particles (Tosi et al., 2020). Hence, DLS is not suitable to provide the accurate size distribution for heterogenous populations. In addition, many EVs have protein corona on their surfaces, which may impact on the accuracy of the results as EVs with and without corona (or unspecific corona) have different hydrodynamic diameters (Varga et al., 2020). Similar to PTA and DLS, nFCM is based on the light scattering from the samples. However, nFCM detects the light scattering from individual nanoparticles passing through the lasers, which prevents the bulk analysis of samples like PTA and DLS. In addition, with the incorporation of multiparameter fluorescence detection, nFCM allows the size detection of individual EVs down to 40 nm (Tian et al., 2018; Gandham et al., 2020). However, the large particles or agglomerates (>200 nm) could not be detected using nFCM since the size distribution of EVs was calibrated based on the size standard of a mixture of silica nanospheres, which was below 200 nm. Finally, we used TRPS with a nanopore NP100, which was calibrated to measure nanoparticles in the range between 50 and 300 nm. Since TRPS detects vesicles within the range of the selected nanopore (Maas et al., 2015), TRPS was less sensitive for detection of EV particles below 50 nm. Size and concentration measurement results showed that each characterization technique has its own limitations due to principles of the measurement, the way samples are prepared, as well as calibration standards (for nFCM and TRPS). Consequently, it led to discrepancies between the results obtained with each of the techniques. These findings highlighted the need to use multiple and complementary techniques to capture the whole population of EVs. Our study also emphasized the need for the careful interpretation of the measurement results, which should account for theoretical and physical principles of each technique, sample preparation, and sample state, which all can impact on the results.

In addition, for the first time, we have demonstrated that large EVs (>100 nm) isolated using ultracentrifugation have a higher dry mass than large EVs isolated using TFF. Here we have introduced the parameter of EV dry mass as a metric to reveal the total mass of molecular cargo inside each vesicle, a measurement not previously made in the EV field. The differences in dry mass between EV isolates suggested different molecular compositions. Indeed, we showed that there were variations between the expression levels of surface markers (CD9, CD63, and CD81) between different EV isolates at sub-population level by using nano-flow cytometry. It has been reported that the presence of each surface marker in EV populations was different depending on the isolation methods of EVs (Tian et al., 2020), which clearly indicated that different subpopulations of EVs have been extracted using different isolation methods. Furthermore, our study showed that the amount of nucleic acid content in EVs isolated using TFF was higher than in EVs isolated using ultracentrifugation for both EV types. The higher nucleic acid content for EVs isolated using TFF was further confirmed at individual vesicle characterization using atomic force nanoscale infrared spectroscopy. By combining multiscale characterization techniques, our study allows robust and precise quantification of molecular composition of EVs. This methodology goes a step beyond conventional characterization methods such as immunoassays or mass spectroscopy, which not only lack single vesicle and subpopulation resolution and flexibility in practical applications but also are very expensive (Trenchevska et al., 2016). This study paves the way to the future diagnostic and therapeutic applications of EVs that depend on identification/quantification of specific cargo.

We have also showed for the first time that the variations in the physicochemical properties of different EV types correlate to their different interactions with cell membranes. By using a distorted grid model to predict how quickly different types of EVs can reach the cell membrane, we demonstrated that the predicted sedimented amount of EVs isolated using ultracentrifugation were 60–100 times higher than EVs isolated using TFF. The calculated higher sedimentation of EVs isolated using ultracentrifugation is likely to be due to the presence of agglomerates, which was consistent with the size distribution and mass results. The agglomerates in EVs isolated using ultracentrifugation were confirmed further using correlative holotomography and fluorescence microscopy. The results of the sedimentation study predict the amount of EVs, which is likely to reach and interact with cells. Depending on the amount of internalized EVs, there may be beneficial or deleterious effects in recipient cells. For example, we showed that DEVs isolated using ultracentrifugation had the highest predicted sedimentation and induced both decreased cell migration and high intracellular NO levels, which indicated an increase in cellular stress post-injury. However, these undesired effects could be associated with EV agglomerates that sedimented rapidly to the cell surface, or with “contaminations” that are typically co-isolated with EV samples during ultracentrifugation. Differences in biological responses to different EV isolates were confirmed using newly developed nitric oxide (NO) probe, which allowed for quantitative and qualitative assessment of intracellular stress (Nasyrova et al., 2015). NO probe enabled us to determine the ability of each EV isolates to promote cell recovery from injury.

In conclusion, we have shown that the physicochemical properties, molecular composition, presence of surface markers, and subsequent biological effects of EVs isolated using ultracentrifugation are markedly different from those isolated by TFF. This study confirms that the isolation method determines which composition of EV sub-populations is isolated. Demonstrated here is the correlation between physicochemical properties and biological effects of EVs, which confirmed that the downstream applications of EVs are determined by the effectiveness of the isolation methods to isolate and fractionate different subpopulations of EVs.

The methodology that we present here for the high-resolution and multiscale measurement of physicochemical and functional properties of EVs is likely to accelerate progress in the development and refinement of isolation methods. Our findings, which uncovered that different EV subpopulations are isolated by different methods, shed new light in our understanding of EV secretion by cells. Furthermore, knowing what the differences in EV composition are depending on the isolation method will support the development of accurate EV-based diagnostic tools for early disease detection. We will also be able to define populations of EVs which have therapeutic potential for specific medical conditions. Taken together, the presented study advanced current understanding of the effect of isolation methods on EV composition and functionality. Our results are likely to contribute to future EV research and provide a backbone for rapid translation of EVs to practical applications.

## Data Availability Statement

The raw data supporting the conclusions of this article will be made available by the authors, without undue reservation.

## Author Contributions

THP, SD, JY, and WC contributed to conception and design of the study. QL, PT, TNP, KL, and MT performed some experiments. VC, ÅJ, M-DD, H-KW, and Y-KC performed the statistical analysis. EN, BK, and PD provided the experimental sources. THP wrote the first draft of the manuscript. All authors contributed to manuscript revision, read, and approved the submitted version.

## Conflict of Interest

The authors declare that the research was conducted in the absence of any commercial or financial relationships that could be construed as a potential conflict of interest.

## Abbreviations

AFM: atomic force microscopy
AFM-IR: atomic force microscope infrared-spectroscopy
CMSC29: chorionic mesenchymal stromal/stem cell line
CEVs: extracellular vesicles derived from chorionic mesenchymal stromal/stem cell
DMSC23: decidual mesenchymal stromal/stem cell line
DEVs: extracellular vesicles derived from decidual mesenchymal stromal/stem cell
EVs: extracellular vesicles
HBSS(-): Hank’s balanced salt solution
LPS: lipopolysaccharide
MSC: mesenchymal stromal/stem cell
PTA: particle tracking analysis
nFCM: nano-flow cytometry
RMM: resonant mass measurement
TRPS: tunable resistive pulse sensing
TFF: tangential flow filtration.
